# Role of DNA Methylation Profile in Diagnosing Astroblastoma: A Case Report and Literature Review

**DOI:** 10.3389/fgene.2019.00391

**Published:** 2019-04-30

**Authors:** Giuseppe Petruzzellis, Iside Alessi, Giovanna Stefania Colafati, Francesca Diomedi-Camassei, Andrea Ciolfi, Lucia Pedace, Antonella Cacchione, Andrea Carai, Marco Tartaglia, Angela Mastronuzzi, Evelina Miele

**Affiliations:** ^1^Department of Paediatric Haematology/Oncology, IRCCS Bambino Gesù Children’s Hospital, Rome, Italy; ^2^Neuroradiology Unit, Department of Imaging, IRCCS Bambino Gesù Children’s Hospital, Rome, Italy; ^3^Department of Laboratories, Pathology Unit, IRCCS Bambino Gesù Children’s Hospital, Rome, Italy; ^4^Genetics and Rare Diseases Research Division, Bambino Gesù Children’s Hospital, IRCCS, Rome, Italy; ^5^Neurosurgery Unit, Department of Neuroscience and Neurorehabilitation, IRCCS Bambino Gesù Children’s Hospital, Rome, Italy

**Keywords:** astroblastoma, DNA methylation profiling, brain tumor, next-generation sequencing, CNS-HGNET-MN1

## Abstract

Astroblastoma is a rare tumor of the central nervous system (CNS) with uncertain clinical behavior. Recently, DNA methylation profiling has been shown to provide a highly robust and reproducible approach for the classification of all CNS tumors across different age groups. By using DNA methylation profiling, a subset of CNS high-grade tumors with astroblastoma-like morphology characterized by the meningioma 1 gene (*MN1*) rearrangements, has been identified; they were termed “CNS high-grade neuroepithelial tumors with *MN1* alteration” (CNS-HGNET-MN1). Here, we describe a case of CNS-HGNET-MN1 diagnosed by DNA methylation profiling, using Illumina Infinium HumanMethylationEPIC BeadChip (EPIC), that offers the opportunity to conduct a brief literature review. The patient presented with an episode of partial seizures involving the right hemisoma. A gross total resection was performed. No other treatment was proposed in light of the histological and molecular findings. After 21 months, the patient is disease-free in good clinical conditions. Also in view of this case, we recommend DNA-methylation profiling as an important tool for diagnosis and more effective patient stratification and management.

## Introduction

Astroblastoma (AB) is one of the rarest tumors of the central nervous system (CNS), with a reported incidence between 0.45 and 2.8% of all primary brain tumors (Navarro et al., 2005). It mostly occurs in infants and young adults, but can be observed also in adulthood (Fu et al., 2013; Merfeld et al., 2018). On imaging studies, the tumor generally appears as a well-circumscribed mass located in the cerebral hemisphere, with heterogeneous contrast-enhancement and, occasionally, cystic areas (Shin et al., 2018).

According to the World Health Organization (WHO), the diagnosis currently rests on the identification of a characteristic morphological pattern of growth, the so called “astroblastic pseudorosettes”: a perivascular distribution of elongated cells containing abundant eosinophilic cytoplasm with a prominent process extending to central hyalinized vessels. Despite such peculiar histopathological characteristics, diagnosis may be challenging, and prognosis is difficult to be accurately estimated. Indeed, the 2016 WHO Classification describes this entity as “other gliomas,” without assignment of any numeric grade, due to its rarity and uncertain clinical behavior (Louis et al., 2016).

Modern molecular approaches are allowing a deeper characterization of AB. Approximately one-third of these tumors harbor the V600E substitution in *BRAF*, like other primarily cortically based, circumscribed gliomas (Lehman et al., 2017). Mutations in isocitrate dehydrogenase (*IDH*) are absent, and no other recurrent mutations have been reported (Fu et al., 2013; Bale et al., 2016; Hirose et al., 2018).

In recent years, DNA methylation profiling has been shown to be a highly robust and reproducible approach for the classification of CNS tumors across age groups (Capper et al., 2018). This technique exploits the notion that the cancer methylome is a combination of both somatically acquired DNA methylation changes and characteristics that reflect both cell of origin and events contributing to transformation (Capper et al., 2018).

By using DNA methylation profiling, Sturm et al. (2016) identified a subset of CNS high-grade tumors with an AB-like pattern characterized by the meningioma 1 gene (*MN1*) rearrangements, described as “CNS high-grade neuroepithelial tumors with MN1 alteration” (CNS-HGNET-MN1). In parallel, *MN1* rearrangements, together with alterations of the X chromosome, have been reported to be a feature of AB (Hirose et al., 2018).

Notwithstanding these insights, AB histology is not specific for any entity, including CNS-HGNET-MN1, and additional genetic characterization is required for a more accurate AB classification. Of note, recent studies have documented methylation profiles and genetic mutations indicating a heterogeneous landscape, which is likely to explain the clinical unpredictability of AB (Wood et al., 2018).

Here, we report a case of parietal tumor with characteristic histological features of AB, confirmed and further classified by DNA-methylation profiling.

## Case Report

**FIGURE 1:**
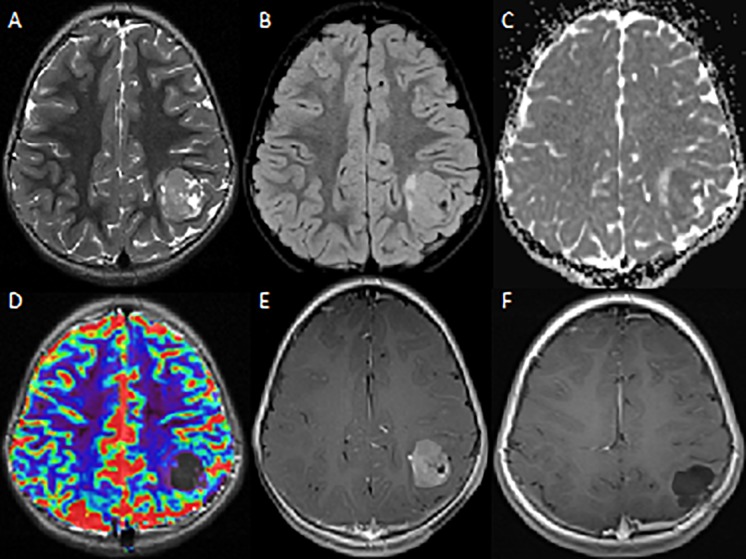
MRI – Axial images. **(A)** T2W image shows a heterogeneous mass in the left parietal lobe with characteristic multicystic bubbly appearance. **(B,C)** There are mild peritumoral edema on FLAIR image and diffusion restriction on ADC. **(D)** No sign of hyperperfusion on dynamic susceptibility-weighted MRI. **(E)** The T1W post-contrast image shows strong contrast-enhancement of the lesion. **(F)** Post-contrast-enhanced cerebral MRI performed 20 months after surgery shows no evidence of recurrent/residual disease.

A four-year-old female was referred to our hospital for the management of a partial seizure, involving the right hemisoma and associate with sialorrhea. In the post-critical time, she had difficulty with speech. An electroencephalogram (EEG), made in the emergency department, evidenced sharp peeks in the left parietal-occipital lobe. She was transferred to our department, where magnetic resonance (MR) was performed as part of diagnostic work up; the radiological investigation revealed a left parietal cortical-subcortical lesion, extending to the white matter; tumor size was 3 cm. (Figure 1).

**FIGURE 2:**
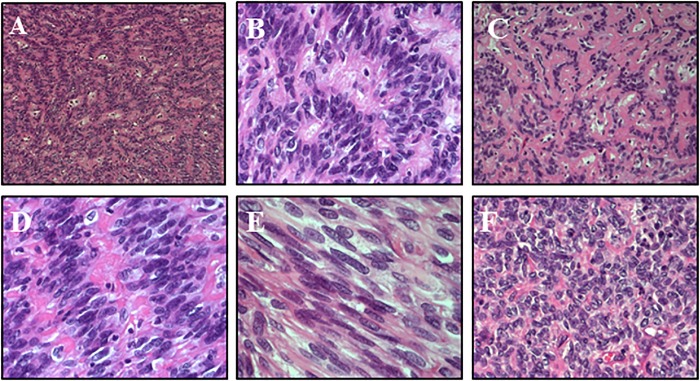
Histology – The tumor was composed of elongated cells with abundant eosinophilic cytoplasm and tapered processes, radiating from the vessels and forming characteristic astroblastic rosettes throughout (**A**: HE 10x, **B**: HE 20x). Sclerosing vessels with scattered foamy histiocytes were present (**C**: HE 20x). Focally, ribbon-like (**D**: HE 40x) or fusiform patterns were observed (**E**: HE 40x). A few highly cellular areas with moderate cellular pleomorphism were noticed (**F**: HE 20x).

Gross total resection was performed. Histologically (Figure 2), the tumor showed non-infiltrative borders and consisted of elongated tapering cells, with abundant eosinophilic cytoplasm, oval nucleus and unconspicuous nucleolus. Astroblastic pseudorosettes were observed throughout; sclerosing vessels with foamy perivascular histiocytes were present. Focally, ribbon-like or fusiform patterns were observed. Few high-cellular areas with moderate cellular pleomorphism were noticed. Immunohistochemistry revealed strong positivity for glial fibrillary acidic protein (GFAP) and OLIG2, mild dot-like and superficial positivity for epithelial membrane antigen (EMA), and negativity for synaptophysin (SYP) and cytokeratin. Proliferation index resulted about 3%; in more dense cellular areas, it reached 7–8%. According to the WHO 2016 classification, the diagnosis of low-grade AB was made. Indeed, High-grade ABs were characterized by multiple foci of high cellularity, anaplasia, increased mitotic activity (>5 mitoses per HPF), elevated proliferative index (>10%), necrosis and microvascular proliferation.

## Methylation Profiling

DNA methylation profiling was performed, following protocols approved by the institutional review board after written consent was obtained from the patient’s parents. Tumor areas with highest tumor cell content (≥70%) were selected for DNA extraction. Samples were analyzed using Illumina Infinium HumanMethylationEPIC BeadChip (EPIC) arrays according to the manufacturer’s instructions, on Illumina iScan Platform. In detail, 500 ng DNA was used as input material for fresh-frozen tissue. Generated methylation data were compared with the Heidelberg brain tumor classifier (Capper et al., 2018) (see text footnote 1) to assign a subgroup score for the tumor compared to 91 different brain tumor entities.

**FIGURE 3:**
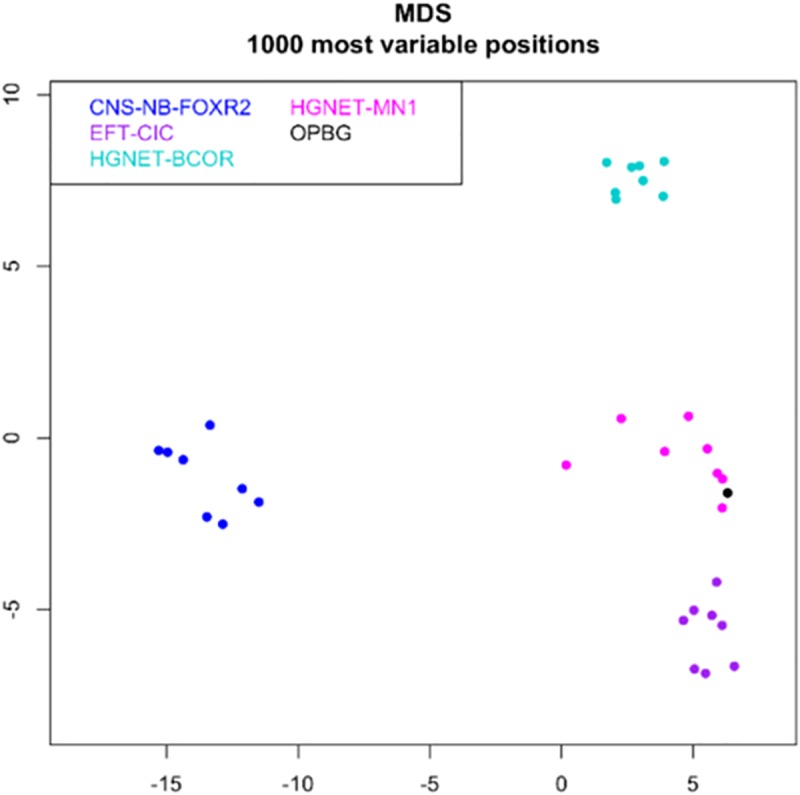
MDS (multidimensional scaling) analysis performed on the 1000 most variable probes of the whole genome DNA methylation data shows a close similarity between our case (OPBG) and CNS-HGNET-MN1, while it clearly separates from other CNS-HGNET. Color legend of the MDS plot as follows: Astroblastoma case (black); CNS-NB-FOXR2 (blue); EFT-CIC (violet); HGNET-MN1 (pink); HGNET-BCOR (light green).

**FIGURE 4:**
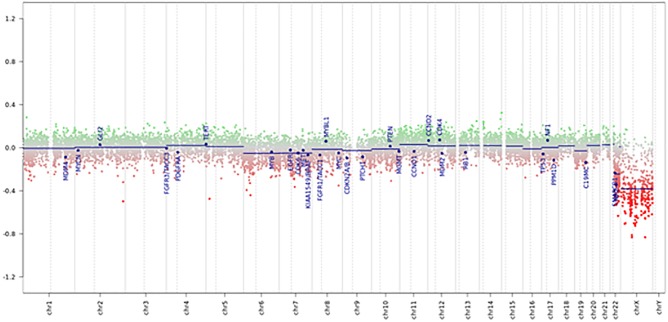
Copy number variation profile – Depiction of chromosome 1 to 22 and X. Gains/amplifications represent positive (green), losses negative (red) deviations from the baseline. 29 brain tumor relevant genomic regions are highlighted. The presented case showed losses of chromosome X and multiple deletions near the *MN1* locus on 22q12.1.

Tumor had a score of 0.99 in the methylation class “CNS high-grade neuroepithelial tumor with MN1 alteration.” Global profiling methylation data were also compared to 32 randomly samples extracted from internal and external datasets (Capper et al., 2018) among those classifying as CNS high grade neuroepithelial tumor with BCOR alteration (HGNET-BCOR), CNS high grade neuroepithelial tumor with MN1 alteration (HGNET-MN1), CNS Ewing sarcoma family tumor with CIC alteration (EFT-CIC), and CNS Neuroblastoma with FOXR2 activation (CNS-NB-FOXR2) (eight samples for each class), using the Heidelberg brain tumor classifier (Capper et al., 2018)^1^. 450 k and EPIC BeadChip data were analyzed by means of R (V. 3.4.4) package minfi (V. 1.24.0), to obtain normalized beta values and to perform Multidimensional scaling (MDS) analysis, following the procedure described by Fortin et al. (2017). Our case displayed global methylation levels close to those of CNS-HGNET-MN1, as evidenced by MDS performed on the 1000 most variable islands in the cohort (Figure 3). Copy number plot showed loss of chromosome X (Figure 4).

In light of the histological and molecular findings, no adjuvant treatment was proposed to the patient.

At the 21-month follow up, the patient is alive, in good clinical conditions, and disease free.

## Discussion

Astroblastoma is a rare glial tumor that was first recognized as a distinct clinicopathological entity by Bailey and Cushing, who coined the term in 1926 (Bailey and Cushing, 1926). In 1930, Bailey and Bucy reported the first series of 25 cases (Bailey and Bucy, 1930). AB is considered a pediatric tumor, but analysis of published reports suggests that age distribution is bimodal, since a prominent peak is observed during childhood (between 5 and 10 years) and a second peak appears to occur in young adulthood (between 21 and 30 years) (Bale et al., 2016). A clear female predominance is reported for all age groups (Hirose et al., 2018).

Clinical onset with signs of raised intracranial pressure is typical for AB, as most cases reported in the literature are characterized by large mass lesions, but non-specific signs and symptoms, including headaches, focal neurological deficits, seizures, nausea, vomiting, diplopia, dizziness and confusion (Navarro et al., 2005).

Radiological analysis of 127 cases evidenced that AB presents with six typical features. Tumor location is supratentorial in the majority (96%) of cases, the most common locations being the parietal and occipital lobes and, less frequently, the temporal and occipital lobes (Cunningham et al., 2016). ABs have been reported as superficial and well-demarcated in 72%, with an extension between 2 and 8 cm. They present as a mixture of cystic and solid components (93%), rarely as a purely solid mass. Contrast enhancement occurs in virtually all cases, often with mild peritumoral edema (80%). On computed tomography ABs present as a hyper-attenuated lesions with macrocalcification in the majority of the cases. On MR imaging, ABs are characteristically dark on T2 sequences and have restricted diffusion (Bailey and Bucy, 1930).

On microscopic examination, AB is characterized by the presence of perivascular psuedorosettes, prominent vascular hyalinization and lack of fibrillary background (Lehman et al., 2017). The tumor may show cellular pleomorphism, vascular proliferation, necrosis, proliferation rate. AB cells are positive for GFAP and OLIG2 and negative for SYP, which support its astrocytic origin. However, the frequent positivity for EMA may suggest an intermediate glial – ependymal phenotype, and a tanycytic origin is also taken into account. The main differential diagnosis is with ependymoma, another common rosette-forming tumor. However, tumor cell processes forming AB pseudorosettes are generally thicker, less tapered, and more distinct (Brat et al., 2000; Lehman, 2008). Moreover, OLIG2 positivity is a useful tool, since ependymoma generally does not express it. Of note, in the present case, diffuse astroblastic pseudorosettes were present, but hyalinization of the vascular network occurred only focally. However, immunohistochemistry profile was consistent with AB.

A clear definition of biological behavior is not well established, owing to the very small number of cases observed. In the 2016 WHO classification, there is no WHO grade, due to the highly variable biological behavior. For the first time, Bonnin and Rubinstein (1989) described two distinct histological types: prognostically favorable “low-grade/well-differentiated” and unfavorable “high-grade/anaplastic.” Prognosis of low-grade ABs is similar to that of low-grade gliomas, instead prognosis of high-grade ABs corresponds to that of anaplastic astrocytoma and has been associated with recurrence and progression. Malignancy relied on the presence of multifocal regions of high cellularity, anaplastic nuclear features, elevated mitotic indices (less than 5 per 10 HPF), vascular proliferation, and necrosis with pseudopalisading. Although most neuropathologists separate these tumors into well-differentiated or anaplastic/malignant forms, there is no universal criterion for making this distinction (Bonnin and Rubinstein, 1989; Brat et al., 2000). Problematically, even within the low- and high-grade categories, the clinical behavior is highly variable (Thiessen et al., 1998; Lau et al., 2006; Ahmed et al., 2014; Janz and Buhl, 2014).

Our case was considered to be in the low-grade group, as it showed an orderly growth pattern with only mild cellular atypia, focal higher proliferation rate, and lack of necrosis or vascular proliferation. A prolonged overall survival has been described for MN1 altered tumors, even in presence of multiple local tumor recurrence (Sturm et al., 2016; Wood et al., 2018).

Brat et al. (2000) analyzed chromosomal abnormalities of seven ABs by Comparative Genomic Hybridization. The most frequent abnormality was gain of chromosome arm 20q. Gain of chromosome 19 was seen in three out of seven, always in concomitance with gain of 20q. Loss of 10p and X, gain of 9q was seen in two cases. These anomalies are different from those of the astrocytic or ependymomas tumors, supporting the thesis that AB is a distinct entity rather than a variant of ependymoma. Fu et al. (2013) noted that AB did not present the typical cytogenetic features of astrocytoma and ependymoma. ABs do not share mutation signatures with low-grade (WHO grade II) diffuse astrocytoma (*IDH1/2* and *TP53*) or ependymomas (*IDH1*, *TP53*, and IDH R132H immunoreactivity).

Next-generation sequencing (NGS) of three cases identified mutations in a few genes known to be altered in low-grade gliomas, (e.g., *BCOR*, *BCORL1*, *ERBB3*, *MYB*, and *ATM)*, but no recurrent mutations were seen (Bale et al., 2016).

In our case, copy number variation (CNV) showed losses of chromosome X and multiple deletions near the MN1 locus on 22q12.1, which is a common event in these tumors (Capper et al., 2018).

By analyzing the DNA methylation profile, our case was classified with an optimal score in the methylation class “CNS high grade neuroepithelial tumor with MN1 alteration.” This methylation class is mainly comprised of tumors with the histological diagnosis of AB, CNS embryonal tumor, NOS and ependymoma. As described, these tumors are usually supratentorial; median age is 16 years (range 5–40). There is a strong predisposition of this tumor to occur in females; in fact, all tumors of the reference cohort were diagnosed in females. Molecularly, most cases harbor a fusion gene involving the transcriptional co-regulator MN1. Most frequent changes in the copy number plot are loss of chromosome X in around 60% of cases (Capper et al., 2018).

Sturm et al. (2016) found that part of CNS high-grade neuroepithelial tumors (CNS-HGNET) had *MN1* alterations, and those tumors showed pseudopapillary patterns and dense pericellular and vascular hyalinization on microscopic examination. They concluded that, histologically, pediatric CNS-HGNET with genetic *MN1* alteration are virtually ABs, raising the possibility that some tumors diagnosed as AB using WHO criteria harbor MN1 alterations. On the other hand, AB histology was not a distinctive feature of CNS-HGNET-MN1, since most cases in this DNA methylation cluster lacked characteristic AB histology (Sturm et al., 2016).

This finding, together with those reported in other available genetic and molecular studies (Hirose et al., 2018; Wood et al., 2018), suggests that “astroblastoma” is a morphological model that can be observed through a spectrum of molecular entities.

Very recently, Wood et al. (2018) further characterized the genetic alterations underlying AB by performing targeted NGS of 500 cancer-associated genes in a series of eight cases and correlating these results with break-apart fluorescence *in situ* hybridization (FISH) analysis of *MN1* locus and DNA methylation profiling. They were able to reclassify most cases into more specific molecular entities: (1) four tumors with *MN1* alteration with good prognosis and mostly classifying as CNS-HGNET-MN1 by DNA methylation profile (3 out of 4); (2) two cases of high-grade astrocytoma originally diagnosed as AB, one harboring *BRAF* pV600E mutation, *CDKN2A/B* deletion and *TERT* promoter mutation, classifying as pleomorphic xantoastrocytoma by DNA methylation; the second characterized by *TP53* mutation and numerous chromosome losses and no specific grouping by DNA methylation profile; (3) two cases of unclassifiable tumors both with intact *MN1* FISH, good prognosis and no clear DNA methylation profile.

Since ABs are rare and tumor description in the literature concerns only individual cases or small collections of cases, optimal treatment protocols have not been established. Whenever feasible, total resection is the best treatment. It provides excellent tumor-control rates. Chemotherapy and radiotherapy could play an adjuvant role. Merfled et al. analysis (Merfeld et al., 2018) identified 63 patients diagnosed with AB between 2004 and 2012. Assigned histopathological grade was known for 38 (60%) patients. Of these, 18 (47.4%) patients were low grade, while 20 (52.6%) patients were high grade. All but one patients were treated with surgical resection. A total of 20 (31%) patients received chemotherapy. Of patients with high-grade tumors, 60% received chemotherapy. Of patients with low-grade tumors, 5.6% received chemotherapy. A total of 26 (43%) patients received radiotherapy. Among patients with high-grade and low-grade tumors, 65 and 17%, respectively, were treated with radiotherapy. That study failed to identify an association between tumor grade and survival, but the authors emphasized that there is no benefit from chemotherapy, even among patients with high-grade tumors. By contrast, among patients with high-grade tumors, patients who did not receive radiotherapy had poor survival.

Mallick et al. (2017) proposed a treatment algorithm for AB. They recommended gross total resection for all these tumors whenever possible. After the initial diagnosis of AB, a central review should be done to reconfirm the diagnosis. As lower-grade tumors behave more indolently, regular follow-up should be preferentially considered for lower-grade AB after gross total resection. Patients with a sub-total excision and those with a high-grade tumor, even if a total excision was performed, should be offered adjuvant radiation along with concurrent temozolomide.

For our patient, considering the gross total resection, the histological and molecular findings, we decided not to propose further treatments.

Patients’ stratification by using DNA-methylation profiling will certainly be a useful tool for guiding the clinical management.

## Conclusion

Astroblastoma is an extremely rare CNS tumor. Morphological diagnosis is difficult, as the typical astroblastic rosettes may be present also in other CNS tumors, including some gliomas and ependymomas. In fact, AB can be considered as a morphologic pattern, which can be associated with a spectrum of molecular entities.

Total resection is the best treatment; the precise role of chemotherapy and radiotherapy is still debated, particularly for high-grade tumors.

We believe that DNA-methylation profiles represents an important instrument for confirming diagnosis, predicting prognosis and better defining the molecular characteristics of AB.

## Ethics Statement

This study was carried out in accordance with the recommendations of the Internal Review Board of the Bambino Gesù Ospedale Pediatrico with written informed consent from all subjects. All subjects gave written informed consent in accordance with the Declaration of Helsinki. The protocol was approved by the Internal Review Board of the Bambino Gesù Ospedale Pediatrico.

## Author Contributions

GP designed the work, structured the study, interpreted the data, and wrote the manuscript. IA acquired the data, and structured and revised the manuscript. GC acquired and elaborated the images. FD-C performed the pathological findings. ACi performed the bioinformatics analysis. LP acquired the data. ACa contributed to patient management and revision. ACar contributed to neurosurgery and revision. MT supervised the study and critically revised the manuscript for intellectual content. AM and EM conceived the idea, structured the study, acquired the data, and wrote, revised, and approved the final version of the manuscript to be published.

## Conflict of Interest Statement

The authors declare that the research was conducted in the absence of any commercial or financial relationships that could be construed as a potential conflict of interest.

## Footnotes

1 http://molecularneuropathology.org

## References

[B1] Ahmed K. A. Allen P. K. Mahajan A. Brown P. D. Ghia A. J. (2014). Astroblastomas: a surveillance, epidemiology, and end results (SEER)-based patterns of care analysis. *World Neurosurg.* 82 291–297. 10.1016/j.wneu.2013.10.035 24141003

[B2] Bailey P. Bucy P. C. (1930). Astroblastomas of the brain. *Acta Psychiatr. Neurol.* 5 439–461. 10.1111/j.1600-0447.1930.tb08230.x

[B3] Bailey P. Cushing H. A. (1926). *Classification of the Tumors of the Glioma Group on a Histogenetic Basis With a Correlated Study of Prognosis.* Philadelphia, PA: J.B. Lippincott Co.

[B4] Bale T. A. Abedalthagafi M. Bi W. L. Kang Y. J. Merrill P. Dunn I. F. (2016). Genomic characterization of recurrent high-grade astroblastoma. *Cancer Genet.* 209 321–330. 10.1016/j.cancergen.2016.06.002 27425854

[B5] Bonnin J. M. Rubinstein L. J. (1989). Astroblastomas: a pathological study of 23 tumors, with a postoperative follow-up in 13 patients. *Neurosurgery* 25 6–13. 10.1227/00006123-198907000-00002 2755581

[B6] Brat D. J. Hirose Y. Cohen K. J. Feuerstein B. G. Burger P. C. (2000). Astroblastoma: clinicopathologic features and chromosomal abnormalities defined by comparative genomic hybridization. *Brain Pathol.* 10 342–352. 10.1111/j.1750-3639.2000.tb00266.x 10885653 PMC8098511

[B7] Capper D. Jones D. T. W. Sill M. Hovestadt V. Schrimpf D. Sturm D. (2018). DNA methylation-based classification of central nervous system tumours. *Nature* 555 469–474. 10.1038/nature26000 29539639 PMC6093218

[B8] Cunningham D. A. Lowe L. H. Shao L. Acosta N. R. (2016). Neuroradiologic characteristics of astroblastoma and systematic review of the literature: 2 new cases and 125 cases reported in 59 publications. *Pediatr. Radiol.* 46 1301–1308. 10.1007/s00247-016-3607-x 27048363

[B9] Fortin J. P. Triche T. J. Hansen K. D. (2017). Preprocessing, normalization and integration of the Illumina human methylation EPIC array with minfi. *Bioinformatics* 33 558–560. 10.1093/bioinformatics/btw691 28035024 PMC5408810

[B10] Fu Y. J. Taniguchi Y. Takeuchi S. Shiga A. Okamoto K. Hirato J. (2013). Cerebral astroblastoma in an adult: an immunohistochemical, ultrastructural and genetic study. *Neuropathology* 33 312–319. 10.1111/j.1440-1789.2012.01351.x 22994361

[B11] Hirose T. Nobusawa S. Sugiyama K. Amatya V. J. Fujimoto N. Sasaki A. (2018). Astroblastoma: a distinct tumor entity characterized by alterations of the X chromosome and MN1 rearrangement. *Brain Pathol.* 28 684–694. 10.1111/bpa.12565 28990708 PMC8028274

[B12] Janz C. Buhl R. (2014). Astroblastoma: report of two cases with unexpected clinical behavior and review of the literature. *Clin. Neurol. Neurosurg.* 125 114–124. 10.1016/j.clineuro.2014.07.013 25108699

[B13] Lau P. P. Thomas T. M. Lui P. C. Khin A. T. (2006). “Low-grade” astroblastoma with rapid recurrence: a case report. *Pathology* 38 78–80. 10.1080/00313020500468871 16484017

[B14] Lehman N. L. (2008). Central nervous system tumors with ependymal features: a broadened spectrum of primarily ependymal differentiation? *J. Neuropathol. Exp. Neurol.* 67 177–188. 10.1097/NEN.0b013e31816543a6 18344909

[B15] Lehman N. L. Hattab E. M. Mobley B. C. Usubalieva A. Schniederjan M. J. McLendon R. E. (2017). Morphological and molecular features of astroblastoma, including BRAFV600E mutations, suggest an ontological relationship to other cortical-based gliomas of children and young adults. *Neuro. Oncol.* 19 31–42. 10.1093/neuonc/now118 27416954 PMC5193018

[B16] Louis D. N. Ohgaki H. Wiestler O. D. Cavenee W. K. (2016). *WHO Classification of Tumours of the Central Nervous System*, 4th Edn. Lyon: International Agency For Research On Cancer.

[B17] Mallick S. Benson R. Venkatesulu B. Melgandi W. Rath G. K. (2017). Patterns of care and survival outcomes in patients with astroblastoma: an individual patient data analysis of 152 cases. *Child’s Nerv. Syst.* 33 1295–1302. 10.1007/s00381-017-3410-5 28477040

[B18] Merfeld E. C. Dahiya S. Perkins S. M. (2018). Patterns of care and treatment outcomes of patients with astroblastoma: a national cancer database analysis. *CNS Oncol.* 7:CNS13. 10.2217/cns-2017-0038 29708401 PMC5977281

[B19] Navarro R. Reitman A. J. de Léon G. A. Goldman S. Marymont M. Tomita T. (2005). Astroblastoma in childhood: Pathological and clinical analysis. *Child’s Nerv. Syst.* 21 211–220. 10.1007/s00381-004-1055-7 15654633

[B20] Shin S. A. Ahn B. Kim S. K. Kang H. J. Nobusawa S. Komori T. (2018). Brainstem astroblastoma with MN1 translocation. *Neuropathology* 38 631–637. 10.1111/neup.12514 30238518

[B21] Sturm D. Orr B. A. Toprak U. H. Hovestadt V. Jones D. T. W. Capper D. (2016). New brain tumor entities emerge from molecular classification of CNS-PNETs. *Cell* 164 1060–1072. 10.1016/j.cell.2016.01.015 26919435 PMC5139621

[B22] Thiessen B. Finlay J. L. Kulkarni R. Rosenblum M. (1998). Astroblastoma: does histology predict biologic behavior? *J. Neurooncol.* 40 59–65. 10.1023/A:1006025000409 9874187

[B23] Wood M. D. Tihan T. Perry A. Chacko G. Turner C. Pu C. (2018). Multimodal molecular analysis of astroblastoma enables reclassification of most cases into more specific molecular entities. *Brain Pathol.* 28 192–202. 10.1111/bpa.12561 28960623 PMC5843525

